# Right Atrial Tumor Invasion: A Rare Presentation of Hepatocellular Carcinoma

**DOI:** 10.7759/cureus.22901

**Published:** 2022-03-06

**Authors:** Kathie Wu, Catherine E Travaline, Leighton Elliot

**Affiliations:** 1 Internal Medicine, Geisinger Medical Center, Danville, USA; 2 Internal Medicine-Pediatrics, Geisinger Medical Center, Danville, USA

**Keywords:** metastasis, portal vein, hepatic vein, right atrial thrombus, hepatocellular carcinoma

## Abstract

Metastatic hepatocellular carcinoma has been associated with a poor prognosis. Common areas of spread include the lungs, portal vein, and portal lymph nodes. Affected patients often have coexisting comorbidities, including cirrhosis, chronic hepatitis, and nonalcoholic fatty liver disease. Our case discusses a rare presentation of hepatocellular carcinoma that had spread to the right atrium, manifesting as acute heart failure in a patient with no history of liver disease. This case highlights the importance of recognizing unusual presentations of advanced hepatocellular carcinoma, as earlier detection can lead to improved patient outcomes.

## Introduction

Hepatocellular carcinoma (HCC) accounts for approximately 90% of primary liver cancers and is the fourth most common cause of cancer death, with an average five-year survival rate of 20% [1]. Affected patient populations are largely those with a history of cirrhosis, chronic hepatitis, and nonalcoholic fatty liver disease. Clinical presentations of HCC vary widely from asymptomatic to nonspecific symptoms of pain, lethargy, encephalopathy, or ascites. Hepatocellular invasion into the hepatic and portal venous system occurs in approximately 10%-40% of patients [2], while tumor invasion into distal larger vascular structures such as the inferior vena cava and the right atrium occurs in approximately 1%-4% of patients [3]. When tumor invasion into cardiac structures occurs, the prognosis is usually extremely poor and can lead to further complications, including embolization [4]. By the time cancer metastasis reaches this extent, treatment options are limited to major surgery such as cardiopulmonary bypass, and efforts are sometimes futile despite these aggressive measures [5]. We discuss a case of hepatocellular carcinoma, which had metastasized to the right atrium and inferior vena cava, presenting as a new-onset heart failure, in a patient with no prior history of hepatitis or cirrhosis. Upon literature review, there are 26 reported cases of cardiac metastasis from hepatocellular carcinoma [6], and of those cases, seven had no confirmed cause of disease. To our knowledge, this case then represents one of the few in literature in which hepatocellular carcinoma with cardiac invasion occurred in a patient without any prior liver disease.

## Case presentation

**Figure 1:**
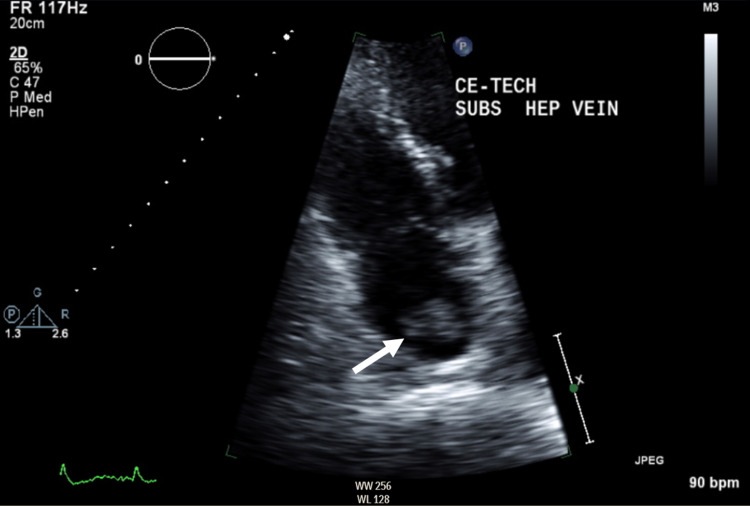
Transthoracic echocardiogram showing the presence of right atrial mass.

**Figure 2:**
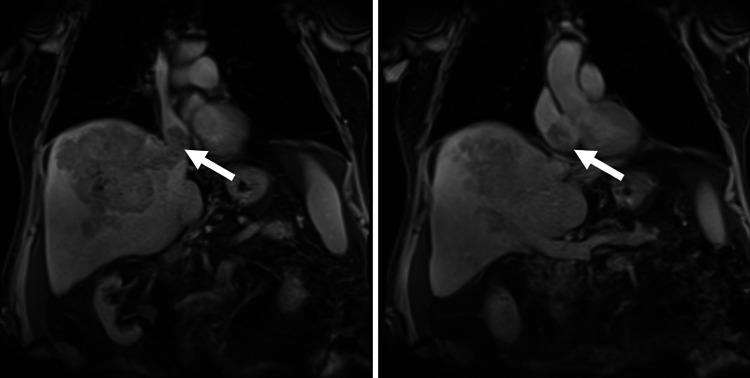
Magnetic resonance imaging (MRI) of the tumor thrombus invading into the right atrium of the heart.

A 71-year-old Caucasian male with a history of hypertension, laryngeal carcinoma status post laser resection and chemoradiation 26 years before presentation, and chronic kidney disease, presented to the hospital with lower extremity edema, abdominal distention, and weight gain. Due to initial concerns for acute heart failure, a transthoracic echocardiogram was obtained, which revealed a large mass in the right atrium, measuring 2.8 cm with origins from the inferior vena cava (Figure 1). Further imaging was obtained with abdominal MRI revealing a 14 x 12 x 11 cm liver mass with thrombus extending into the right portal vein, right hepatic vein, inferior vena cava, and right atrium (Figure 2). The mass showed early arterial phase enhancement, subsequent washout, and residual capsular enhancement consistent with hepatocellular carcinoma. An alpha-fetoprotein level was obtained and elevated to 7,442 ng/mL (normal: 0.0 - 6.7 ng/mL). With MRI findings consistent with HCC and elevated alpha-fetoprotein levels, no further testing was deemed necessary for diagnosis. Viral testing was negative for hepatitis A, B, C, herpes simplex virus (HSV), and cytomegalovirus (CMV), and the patient had no prior history of cirrhosis, so no identifiable risk factor for the development of HCC was identified. Due to the presence of portal invasion, the patient was classified as Barcelona clinic liver cancer (BCLC) Stage C. As the tumor was unresectable at the time of diagnosis, the patient underwent a transatrial stent to alleviate inferior vena cava (IVC) obstruction. He was recommended to undergo Y90 embolization sequenced with systemic therapy with sorafenib, but due to the patient's desire to optimize quality of life, he decided to forego palliative therapy and subsequently died on hospice care shortly thereafter. 

## Discussion

Tumor invasions into the vena cava and right atrium are rare complications associated with hepatocellular carcinoma. Tumor burden may have potential complications of heart failure, pulmonary embolism, and sudden cardiac death. Median survival rates of the localized disease range from 9-33 months with surgical resection. Previously, metastatic disease to the IVC and right atrium were treated with palliative focus, with an average of 2-3 months survival rates [7]. With advancements in technology and surgical techniques, aggressive treatment of metastatic hepatocellular carcinoma to the IVC and right atrium, including systemic chemotherapy, transarterial chemoembolization, intraarterial chemotherapy, radiation, or surgery, can improve overall survival time anywhere from 4.5-30.8 months depending on the treatment modality [7]. In a patient without any known risk factors for hepatocellular carcinoma, this case demonstrates a rare presentation and complication of the disease. In our patient, though he had good performance status and was a candidate for chemoembolization, the concern for intolerable treatment side effect profile ultimately deterred his decision to pursue treatment. Though treatment methods are advancing, they are not without risks and complications, including pulmonary embolism, hepatic abscess, gastric mucosa injury [8]. Since our patient had no prior history of cirrhosis, there was no indication for hepatocellular screening, but earlier detection may have improved outcomes. This case highlights the need for routine screening for HCC in patients with a history of cirrhosis, as earlier stages of HCC can be treated with ablation or resection and may be potentially curable [9].

## Conclusions

Vascular invasions of hepatocellular carcinoma are associated with dismal prognosis. While there are populations with previous risk factors, including hepatitis and cirrhosis, that have a higher chance of developing this disease, hepatocellular carcinoma can still develop in patients without any preexisting risks. This case helps us recognize alternative presentations of HCC that may lead to earlier diagnoses for other patients and speaks to the need for prompt routine screenings in high-risk populations. Given the advancements made in the treatment of hepatocellular carcinoma, including metastatic disease, this can lead to better overall outcomes.
